# Prosthetic Joint Infection in Patients with Rheumatoid Arthritis: An Outcome Analysis Compared with Controls

**DOI:** 10.1371/journal.pone.0071666

**Published:** 2013-08-26

**Authors:** Pang-Hsin Hsieh, Kuo-Chin Huang, Hsin-Nung Shih

**Affiliations:** 1 Department of Orthopaedics, Chang Gung Memorial Hospital, Taoyuan, Taiwan; 2 Department of Orthopaedics, Chiayi Chang Gung Memorial Hospital, Chiayi, Taiwan; 3 College of Medicine, Chang Gung University, Taoyuan, Taiwan; Harvard Medical School, United States of America

## Abstract

**Background:**

Patients with rheumatoid arthritis (RA) have been shown to have an increased susceptibility to the development of prosthetic joint infection (PJI) after hip or knee replacement. However, little information is available on the demographic data, outcome of treatment and prognostic factors in RA patients when compared to those in non-RA patients.

**Methods/Principal Findings:**

We performed a retrospective cohort analysis of all cases of PJI that were treated at our institution between 2002 and 2008. Of 346 episodes of PJI during the study period, 46 (13.3%) occurred in patients with RA. Compared to the non-RA cohort, RA patients with PJI were female predominant (74% vs 27%, p<0.001), younger (median age, 51 vs 63 years, p<0.001) and developed infection earlier (median joint age, 72 vs 128 days, p<0.001). The 2-year survival rate free of treatment failure was lower in RA patients with PJI episodes either treated with débridement (22% vs 52%, p = 0.002) or two-stage exchange (78% vs 95%, p = 0.004). A longer duration of symptoms before débridement surgery (median, 11 vs 5 days, p = 0.015), and absence of antibiotics in bone cement for two-stage exchange (relative risk, 8.0; p = 0.02) were associated with treatment failure in patients with RA.

**Discussion:**

The outcome of PJI in RA patients was generally worse than that in non-RA patients. Risk of treatment failure increased in the setting of delayed débridement and two-stage exchange without the use of antibiotic-impregnated bone cement. These findings highlight the importance of vigilant monitoring and aggressive treatment for PJI in RA patients.

## Introduction

Increased evidence reveals that patients with rheumatoid arthritis (RA) are at a high risk for developing several comorbid disorders, that these conditions may have atypical features and thus may be difficult to diagnose and treat [1]. Like other inflammatory disorders, RA appears to significantly increase the risk for bacterial, tubercular, fungal, and viral infections, with all these infections being more common in more active and severe RA [1], [2]. Infection is also one of the most devastating complications following prosthetic joint replacement (PJR), a common orthopaedic procedure that is often required in patients with RA. Studies have shown that RA is an independent risk factor for postoperative prosthetic joint infection (PJI) [3]–[5]. Patients with underlying RA are at 1.8- to 4-fold higher risk of PJI when compared to those who undergo total hip arthroplasty (THA) or total knee arthroplasty (TKA) for other etiologies [3.4]. In addition, the immunosuppressive medications frequently used in the treatment of RA may increase the risk further [2].

Treatment for PJI can be divided into two main groups: prosthesis removal with subsequent reimplantation (two-stage exchange), or débridement and implant retention with antibiotic therapy. Removal of prosthesis carries a higher chance to eradicate infection but requires extensive surgery and often prolongs immobilization [6]. Débridement and retention is an attractive alternative because this less-extensive surgery is associated with a lower probability of procedure-related morbidity [7]. The main problem of débridement, however, is that a substantial number of patients will ultimately experience a relapse of infection, necessitating further surgery [6], [7]. The risk of treatment failure for these procedures is influenced by a number of factors, including the underlying immunocompromised comorbidity [7], [8].

Despite numerous reports on the treatment of PJI, little is known from the literature regarding the outcome of PJI in patients with RA. Most previous studies included only a small number of RA patients [7]–[12], ranging from 3 to 15 cases in studies of sizes ranging from 24 to 168 patients. One recently published study reported the results from the largest case series of PJI in 160 RA patients [13]. However, the study was conducted on patients treated between 1969 and 1995. Treatment of RA and PJI changed substantially over such a long study period. Outcomes of treatment at present may differ significantly from those of these early cohorts because of the utilization of modern approaches for RA, including the disease modifying anti-rheumatic medication (DMARD) and the biologic drugs, and more sophisticated surgical measures for PJI, such as antibiotic-impregnated bone cement. Most importantly, previous studies did not include a control group of patients without RA, hampering comparison of outcome of PJI between the RA and non-RA cohorts.

Based on strict case definitions for RA and PJI, we aimed to determine the demographic characteristics of RA patients who developed PJI following THA and TKA. We also explored the cumulative survival rates free of treatment failure in a modern cohort of patients with underlying RA and compared them to those of patients without RA but underwent the same type of surgery. In addition, we intended to identify potential risk factors leading to treatment failure in RA patients with PJI.

## Materials and Methods

### Study population

This retrospective cohort study included all patients with a diagnosis of hip or knee PJI who were treated at our institution between January 2002 and December 2008. After obtaining approval from the Institutional Review Board, cases were identified by matching the International Classification of Diseases (ICD), 9th Revision code specific for PJI (996.66) in the computerized joint replacement registry database of the hospital. The medical records were reviewed and confirmed by two independent researchers (PHH and HNS). The patients were observed from the date of diagnosis of PJI until evidence of treatment failure, final clinic visit, death, or loss to follow-up.

### Definitions

#### PJI

PJI was defined as isolation of the same microorganism from at least two cultures of joint aspirates or intraoperative tissue specimens or isolation from at least one intraoperative culture of microorganisms plus evidence of infection at the site of hip or knee prosthesis: presence of a discharging sinus, operative findings of purulence, or positive laboratory and histopathological tests [14].

#### RA

RA was defined only if the patients met the American College of Rheumatology (ACR) classification criteria [15]. If chart review could not provide sufficient information for the verification of diagnosis, the treating rheumatologist was contacted to confirm the diagnosis.

#### Perioperative immunosuppressive medication

Perioperative immunosuppressive medication was defined as the use of any of the following medication within 2 weeks before and after surgery: 1) steroid, 2) DMARD, including methotrexate, azathioprine and leflunomide, and 3) biologic response modifiers, including anti-tumor necrosis factor (anti-TNF) agents and anakinra.

#### Joint age

Joint age was defined as the duration between the index joint replacement surgery and the development of PJI.

#### Persistent infection

Persistent infection was defined as the occurrence of PJI at any time after the initial surgery due to the same microorganism isolated at the time of original débridement.

#### Recurrence of infection

Recurrence of infection was defined as any PJI that occurred after completion of a staged prosthetic removal and reimplantation surgery, regardless of the causative microorganism.

#### Treatment failure

Treatment failure was defined as the occurrence of the following conditions at any time after the initial surgical treatment: 1) persistent infection, 2) recurrence of infection, 3) development of a sinus tract, 4) amputation, or 5) death related to PJI.

We summarized the collected data at the time of study enrollment, which included demographic data of the patients, duration of RA, surgical history of the joint, presenting signs and duration of PJI, surgical and medical treatments and bacteriologic results. They were analyzed and compared between RA and non-RA patients to determine demographic characteristics, treatment outcome and prognostic factors predicting treatment failures.

### Statistical Analysis

A Chi-square analysis or a Fisher’s exact test was used where appropriate for analyzing categorical data. For numerical data, independent *t*-test or the nonparametric Mann-Whitney *U* test were utilized for between-group comparisons. The survival rate free of treatment failure was estimated using the Kaplan-Meier survival method and the log rank test. Statistical significance was defined as *p*<0.05. All statistics were two-sided and performed using SAS software (version 9.1.3, SAS Inc., Cary, NC).

### Ethic statement

The datas were analyzed after approval by the ethic committee (Institutional Review Board) of the Chang Gung Memorial Hospital in Taiwan. We did not obtain informed consent from the patient due to a statement of this committee, that analyzing patient data retrospectively requires no informed consent.

## Results

### Study population

Forty-six episodes of PJI, occurring among 43 patients from 1 January 2002 through 31 December 2008, were treated at our institution. This accounted for 46 (13.3%) of 346 first-time episodes of culture-proven PJI encountered during the study period (Table 1). In the same period of time, the median age, M/F ratio and infection rate of patients undergoing total joint arthroplasty, with and without RA, were 48 years vs. 62 years, 0.21 vs. 0.64, and 0.98% vs. 3.20%, respectively. Compared to the non-RA cohort, RA patients with PJI were female predominant (74% vs. 27%, p<0.001), younger (median age, 51 vs. 63 years, p<0.001) and developed infection earlier following the index joint replacement surgery (median joint age, 72 vs. 128 days, p<0.001). Although the difference did not reach statistical significance, patients with RA were more likely to develop infections caused by multiple microorganisms (polymicrobial PJI; 11% vs. 4%, p = 0.06), and in the setting of revision surgery (44% vs. 32%, p = 0.14). The two groups did not considerably differ in joint location, history of diabetes, presentations of PJI and the type of initial surgical treatment modalities.

**Table 1:** Characteristics among RA and non-RA patients with PJI.

Variable	RA (n = 46)	Non-RA (n = 300)	*P* Value
**Age**, median years (ranges)*	51 (35–75)	63 (32–85)	<0.001
**Female gender** *	34 (74)	82 (27)	<0.001
**Total knee arthroplasty**	13 (28)	91 (30)	0.78
**Joint age**, median days (range)*	72 (8–478)	128 (6–976)	<0.001
**Revision prosthesis**	20 (44)	97 (32)	0.14
**Microbiology**			
***Staphylococcus aureus***	25 (54)	184 (61)	0.37
Gram-negative organisms	6 (13)	47 (16)	0.65
Polymicrobial	5 (11)	13 (4)	0.06
**Diabetes mellitus**	15 (33)	114 (38)	0.48
**Presentation of infection**			
Discharging sinus	17 (37)	107 (36)	0.87
Purulent fluid or pus in the joint	18 (39)	103 (34)	0.53
Fever (temperature ≥38.3°C)	5 (11)	38 (13)	0.73
Bacteremia	4 (9)	15 (5)	0.31
**Initial surgical treatment**			
Débridement	21 (46)	133 (44)	0.87
Removal of prothesis	25 (54)	167 (56)	0.87

**NOTE**. Data are no. (%) of joints, unless otherwise indicated.

* Statistical significance (p<0.05).

### Microbiologic findings

The microbiologic findings of the 46 episodes of PJI in patients with RA are shown in Table 2. *Staphylococcus aureus,* the most commonly isolated pathogen, was involved in 25 episodes (54%), followed by coagulase-negative staphylococcus in 4 (9%) episodes and *Pseudomonas aeruginosa* in 4 (9%) episodes.

**Table 2:** Microbiologic findings of 46 episodes of PJI in RA patients treated between 2002 and 2008.

Infecting Microorganisms	Value
***Staphylococcus aureus***, methicillin-resistant	16 (35)
***Staphylococcus aureus***, methicillin-sensitive	9 (19)
***Staphylococcus***, coagulase-negative	4 (9)
***Pseudomonas aeruginosa***	4 (9)
***Streptococcus spp***.	2 (4)
***Enterococcus spp***.	2 (4)
***Escherichia coli***	1 (2)
***Bacteroid spp***.	1 (2)
***Peptostreptococcus spp***.	1 (2)
***Klebsiella pneumoniae***	1 (2)
Polymicrobial	5 (11)

**NOTE**. Data are no. (%) of joints.

Because of rounding, percentages may not add to 100%.

### Treatment and Outcomes

**Figure 1:**
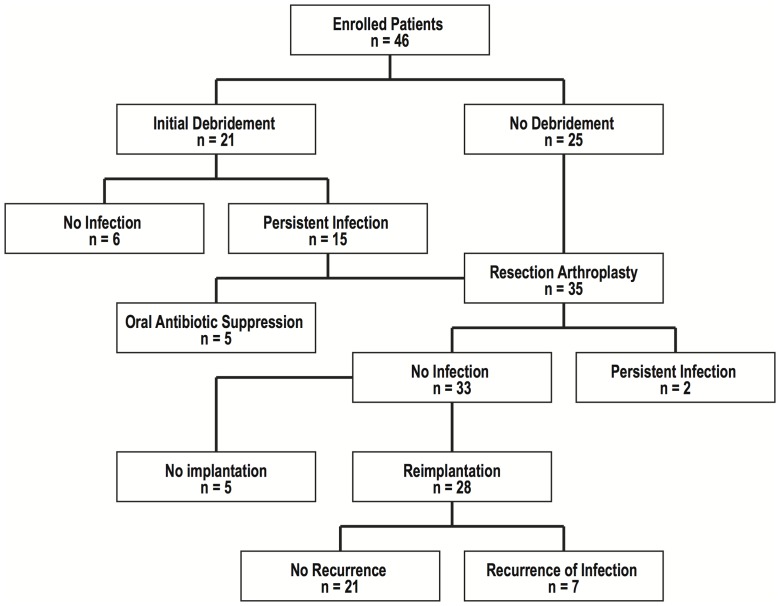
Flow chart of treatment modalities for 46 episodes of PJI in RA patients treated between 2002 and 2008.

The number of PJI episodes in RA patients treated with each therapeutic modality is outlined in Figure 1. Débridement and retention of the prosthesis was the initial treatment for 21 episodes (46%), which typically included complete exposure of the joint and removal of the inflamed soft tissues and bone. Evidence of persistent infection was noted in 15 (71%); 10 of them underwent further surgery to remove the prosthesis (resection arthroplasty), and 5 of them were treated with long-term antibiotic suppression without additional operations.

A total of 35 (76%) patients required resection arthroplasty to control PJI. No evidence of infection was seen in 33 (94%) of them. Two patients (6%) had persistent infection despite prosthesis removal, repeated débridement and prolonged antibiotic therapy. At the time of writing this paper, one patient had amputation to control sepsis and the other was hospitalized due to a draining wound.

Five patients in whom PJI was eradicated by resection arthroplasty did not undergo reimplantation. Seriously compromised medical conditions and extensive bone and soft tissue loss prohibited a stable reimplantation, which were reasons preventing further surgery in these patients.

Twenty-eight patients underwent prosthesis reimplantation after a median period of 103 days (range, 92–132 days) following prosthesis removal. Of these, PJI recurred in 7 patients (25%), occurring at a median of 16 months (range, 6–35 months) after reimplantation. The infecting microorganism isolated from recurrent cases was the same as that isolated from original PJI in 4 patients and different in 3 patients.

### Survivorship and prognostic factors

**Figure 2:**
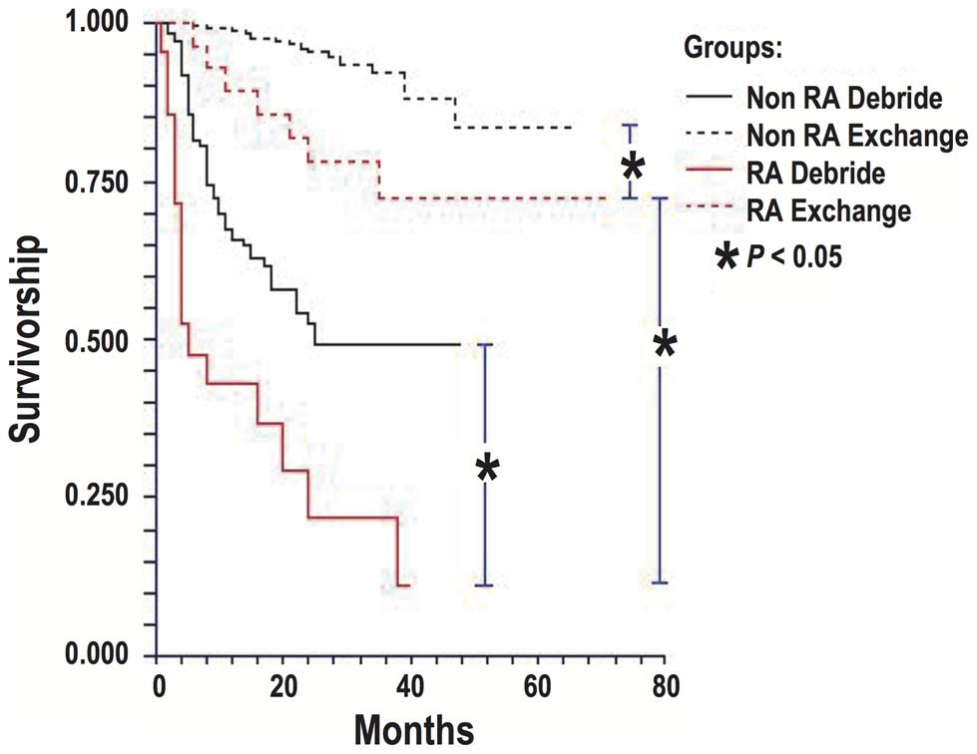
Survival free of treatment failure among episodes of PJI treated with débridement and two-stage exchange from 2002 through 2008. Debride: débridement and retention of prosthesis; Exchange: Two-stage exchange arthroplasty.

The 2-year survival rate free of treatment failure for PJI in RA patients was 22% (95% confidence interval [CI], 14%–34%) for débridement and retention, and 78% (95% CI, 66%–83%) for two-stage exchange (Fig. 2). RA patients with PJI who underwent débridement alone were significantly more likely to experience treatment failure than those who underwent two-stage exchange (p<0.001). The outcomes of treating PJI in RA patients were generally worse than those of PJI in non-RA patients treated with similar surgical modalities, for the latter the 2-year estimates of survival free of treatment failure was 52% (95% CI, 39%–62%; p = 0.002) for débridement and retention, and 95% (95% CI, 85%–100%; p = 0.004) for two-stage exchange.

The potential prognostic factors leading to treatment failure in RA patients receiving débridement and retention are outlined in Table 3. A longer duration of symptoms prior to débridement surgery (median, 11 vs 5 days, p = 0.015) was associated with treatment failure. Age, gender, duration of RA, history of diabetes, joint age, revision prosthesis, inflammatory markers, duration of antibiotic therapy, perioperative use of immunosuppressive medication, presenting symptoms of PJI and the infecting microorganisms were not found to be risk factors with statistical significance.

**Table 3:** Selected variables in 21 RA patients with PJI treated with débridement and retention of the prosthesis.

Variable	Successful Débridement (n = 5)	Persistent Infection (n = 16)	*P* Value
**Age**, median years (ranges)	49 (43–70)	55 (37–65)	0.59
**Male gender**	2 (40)	2 (13)	0.23
**Total knee arthroplasty**	2 (40)	5 (31)	NS
**Duration of RA**, median years (range)	16 (7–27)	17 (9–32)	0.56
**Joint age**, median days (range)	66 (39–438)	82 (9–378)	0.80
**Duration of symptoms**, median days (ranges)*	5 (3–8)	11 (4–64)	0.015
**Revision prosthesis**	2 (40)	9 (56)	0.64
**Laboratory data**			
CRP (mg/L), median (range)	39 (19–94)	34 (14–178)	0.48
ESR (mm/hr), median (range)	77 (43–147)	59 (31–128)	0.15
**Microbiology**			
***Staphylococcus aureus***	3 (60)	8 (50)	NS
Gram-negative organisms	1 (20)	3 (19)	NS
Polymicrobial	0 (0)	3 (19)	0.54
**Preoperative medications**			
Steroid	3 (60)	12 (75)	0.59
Other immunosuppressive medications	2 (40)	12 (75)	0.28
**Intravenous antibiotic therapy**, median days (range)	32 (24–47)	36 (14–49)	0.63
**Oral antibiotic suppression**, median days (range)	54 (35–98)	50 (32–104)	0.72
**Diabetes mellitus**	2 (40)	5 (31)	NS
**Presentation of infection**			
Discharging sinus	0 (0)	7 (44)	0.12
Purulent fluid or pus in the joint	1 (20)	4 (25)	NS
Fever (temperature ≥38.3°C)	0 (0)	2 (13)	NS
Bacteremia	0 (0)	2 (13)	NS

**NOTE**. Data are no. (%) of joints, unless otherwise indicated.

NS: Not significant; CRP: C-reactive protein; ESR: erythrocyte sedimentation rate.

* Statistical significance (p<0.05).

The univariate analysis of RA patients undergoing two-stage exchange arthroplasty is presented in Table 4. Regarding the duration of the evolution of symptoms in patients undergoing two-stage exchange, there were no differences between patients with and those without treatment failure (recurrent infection). Patients in whom antibiotic-impregnated bone cement was not used in reimplantation surgery had a significantly increased risk of recurrent PJI (relative risk, 8.0; p = 0.02). No other risk factor was identified in this study.

**Table 4:** Selected variables in 28 RA patients with PJI treated with two-stage exchange.

Variable	Recurrent Infection (n = 7)	No Recurrent Infection (n = 21)	*P* Value
**Age**, median years (ranges)	55 (37–65)	48 (35–73)	0.81
**Male gender**	2 (29)	5 (24)	NS
**Total knee arthroplasty**	3 (43)	5 (24)	0.37
**Duration of RA**, median years (range)	17 (8–22)	12 (6–28)	0.36
**Duration of symptoms**, median days (range)	12 (4–45)	10 (3–52)	NS
**Diabetes mellitus**	2 (29)	4 (19)	0.62
**Laboratory data**			
CRP (mg/L), median (range)	12 (8–27)	16 (6–30)	0.80
ESR (mm/hr), median (range)	43 (19–103)	39 (21–130)	0.67
**Preoperative medications**			
Steroid	2 (29)	8 (38)	NS
Other immunosuppressive medications	4 (57)	6 (29)	0.20
**Surgical factors**			
Initial débridement	1 (14)	7 (33)	0.63
Interim period, median days (range)	103 (92–132)	89 (78–157)	0.89
Operation time, median minutes (range)	115 (85–160)	100 (70–155)	0.33
Use of antibiotic-impregnated bone cement*	1 (14)	15 (71)	0.02

**NOTE.** Data are no. (%) of joints, unless otherwise indicated.

NS: Not significant; CRP: C-reactive protein; ESR: erythrocyte sedimentation rate.

* Statistical significance (p<0.05).

## Discussion

PJR is a salvage procedure for patients with RA, a chronic debilitating inflammatory arthritis that progressively destroys joint structures. It has been estimated that up to 24% of patients with early RA will eventually require first major joint replacement 16–20 years after the diagnosis [16], and 5–7% of all patients undergoing THA or TKA have underlying RA [5]. Despite improvements in operative techniques, aseptic procedures, and the use of perioperative antibiotic prophylaxis have reduced the risk of infection to around 1% in general patients undergoing THA and TKA [17], this devastating complication remains a serious threat to RA patients, observed at an incidence of 3.1–4.2% [3], [4].

To the best of our knowledge, this study is the first to report on the outcomes of PJI in patients with RA who have undertaken THA and TKA, and compare them to those of PJI without RA. Our data suggest that PJI in RA patients represents a substantial proportion (13.3%) of all PJI occurrences, and patients with RA tend to develop PJI earlier than non-RA patients after joint replacement surgery. *Staphylococcus aureus* remains the most common causative pathogen for PJI in RA patients, which is similar to that in non-RA cohort. Despite not reaching statistical significance, there seems to be a tendency for more polymicrobial PJIs in RA patients (p = 0.06). These findings underline the importance of selecting a broader perioperative antimicrobial prophylaxis for RA patients and more careful monitoring of infection, especially in the early postoperative period.

The choice between retention and removal of the prosthesis for the management of PJI is a challenging one. The treating clinicians and patients are often forced to confront a compromise between short-term surgical morbidity and mortality and long-term recurrence of infection. In one study of 99 episodes of PJI, débridement and retention of prosthesis was associated with a favorable 2-year survival rate of successful treatment of 60%, and RA was not identified as risk factor for treatment failure [7]. These observations, however, were based on a mixed case cohort including only a small number of RA patients (6; 6%), and may thus lack sufficient power to detect the difference. In the current study, the outcome of débridement and retention in RA patients was significantly worse than that in non-RA patients. We also observed a significantly inferior 2-year survival rate free of treatment failure when RA patients were treated with débridement rather than two-stage exchange (22% vs. 78%, p<0.001). Our findings were consistent with those of Berbari et al. [13], who found that PJI in RA patients treated with débridement and retention had a 5.9-fold increased risk of treatment failure, compared to joints treated with two-stage exchange arthroplasty. Removal of the infected prosthesis allows for thorough débridement and complete eradication of the pathogens, whereas débridement and retention of the prosthesis usually does not allow complete eradication of the biofilm present on the prosthesis, accounting for the high rate of recurrence associated with this surgical procedure. We, therefore, recommended that the initial attempt to preserve the implant may be deleterious to treatment outcome, particularly in RA patients.

Despite the dismal figure associated with débridement and retention in our series, a request by these debilitated patients to retain the joint prosthesis at any cost is a common clinical scenario. In fact, only 5 (24%) out of 21 patients had a successful attempt. A short duration of symptoms before surgery was the only identified factor contributing to the success, and in all successful cases the surgery was performed within 8 days after the onset of symptoms. This is consistent with outcome data from other cohorts of patients. Marculescu et al. [7] reported that delayed treatment of ≥8 days after the onset of symptoms are linked with a significantly increased risk of treatment failure following débridement and prosthesis retention. Tattevin et al. [18] also found a significantly short interval (<5 days) from onset of symptoms to débridement in patients who were successfully treated compared to those who failed. These results clearly indicated that débridement alone should not be performed for patients who had prolonged symptoms as a failed attempt incurs the surgical and anesthesia morbidity in RA patients because further surgery is often required.

Other factors, such as the presence of a sinus tract and joint age, have been associated with failure in débridement [7], [19]. In our current study, given the small number of successful débridement in patients with RA, we were unable to identify other poor prognostic factor. Nevertheless, it is important to note that none of the PJI episode in RA patients that was managed successfully had had a sinus tract.

The current trend of treatment for PJI is two-stage exchange arthroplasty. The established protocol in our institution, therefore, does not include single-stage exchange arthroplasty due to its unreliable clinical results [17]. In our study, RA patients with PJI episodes treated with two-stage exchange had a risk of another infection of 25%, as compared with 5% in non-RA patients. Our finding is similar to that of Berbari et al. [13], who found the rate of reinfection of 21% in RA patients undergoing two-stage resection and reimplantation for PJI. The rate of recurrent infection for two-stage procedure in a mixed patient cohort is typically 4–9% [8], [9], [11]. The reason for the higher recurrent rate in RA patients is not clear. Possible explanations include the intrinsic disease severity with associated higher infection susceptibility, the use of steroid and other immunosuppressive medications, or the reinfection is simply due to persistence of bacteria in the joint and a low sensitivity of the diagnostic methods used to identify their presence prior to reimplantation [3]. Data provided in this study here do not give a clear explanation, so further investigation is warranted to elucidate this.

The use of antibiotic-loaded bone cement at reimplantation carries a lower incidence of recurrent infection. This finding supports the concept that the initial burst of antibiotics released is adequate to prevent the formation of bacterial biofilm on the implant and hence to prevent a postoperative infection. Our result is also consistent with that of a randomized clinical trial and a recent large registry-based study which support a role of antibiotic-loaded cement for infection prophylaxis, especially in the revision setting or in patients with risk factors of infection [4], [20]. Although we did not regularly use the antibiotic-loaded cement in PJR during the study period between 2002 and 2008, we start to consider routinely using the antibiotic-impregnated bone cement in two-stage exchange arthroplasty for RA patients with previous PJI after finishing the current study.

In evaluation of the influence of perioperative immunosuppressive medication on treatment failure, we have excluded agents that are of lower immunosuppressive potency (such as hydroxychloroquine) and focused the analysis on those with higher immunosuppressive potency (such as methotrexate). Our results showed that perioperative use of these agents did not increase failure rates in patients with PJI and RA treated with débridement or two-stage exchange. Data from prospective studies generally support the continuing methotrexate use during the perioperative period, demonstrating that this practice does not increase surgical infection [21], [22]. However, data on other DMARDs or biologic drugs are surprisingly sparse.

Our study has several limitations. First, this is a retrospective study harboring all the potential drawbacks implicit in such a study design. Although our institution is a tertiary care referral center with established protocol for the treatment of PJI and in all cases the decisions were made by consultation with the infection specialists, there remains a potential of uncontrolled selection bias among treating physicians. In addition, the number of patients with RA included during the study period was relatively small, and thus the study may have lacked power to detect slight differences among subsets of patients. Finally, the quality of surgical procedures among surgeons and patients is likely to affect the outcome. However, it was very difficult to evaluate the quality of surgery and thus we did not address this variable specifically in the present study.

In summary, RA is a common comorbidity of PJI in our institution, observed in 13.3% of all PJI episodes. PJI in RA patients tend to develop in the early postoperative period. We found that outcome of PJI treatment in RA patients was generally worse than that in non-RA patients. Retention of the prosthesis by débridement alone should not be attempted in the setting of delayed treatment. Two-stage exchange with the use of antibiotic-impregnated bone cement was associated with lowest rate of treatment failure. These findings highlight the importance of vigilant monitoring and aggressive treatment for PJI in RA patients.
